# Assessment of prescribing patterns of antibiotics using National Treatment Guidelines and World Health Organization prescribing indicators at the Ghana Police Hospital: a pilot study

**DOI:** 10.11604/pamj.2021.39.222.29569

**Published:** 2021-08-02

**Authors:** Thomas Opoku Darkwah, Daniel Kwame Afriyie, Jacqueline Sneddon, Alison Cockburn, Mercy Naa Aduele Opare-Addo, Benjamin Tagoe, Seth Kwabena Amponsah

**Affiliations:** 1 Department of Pharmacy, Ghana Police Hospital, Accra, Ghana,; 2 Scottish Antimicrobial Prescribing Group, Healthcare Improvement Scotland, Glasgow, United Kingdom,; 3 National Health Service (NHS) Lothian Antimicrobial Management Team, Western General Hospital, Crewe Road South, Edinburgh, United Kingdom,; 4 Department of Pharmacy Practice, Faculty of Pharmacy and Pharmaceutical Sciences, College of Health Sciences, Kwame Nkrumah University of Science and Technology, Kumasi, Ghana,; 5 Department of Medical Pharmacology, University of Ghana Medical School, Accra, Ghana

**Keywords:** Antimicrobials, antimicrobial resistance, infections, prescription pattern, WHO “aware” classification

## Abstract

**Introduction:**

irrational or inappropriate prescribing of antibiotics is a major problem in healthcare and leads to antibiotic resistance. There is the need to understand the prescribing patterns and antibiotic stewardship in health facilities to support appropriate antibiotic use. A study was carried out to evaluate prescribing pattern of antibiotics at the Ghana Police Hospital using National Standard Treatment Guidelines (STG) and World Health Organization (WHO) prescribing indicators.

**Methods:**

a cross-sectional descriptive study was conducted at the Ghana Police Hospital. Data on prescriptions of antibiotics for both out-patients and in-patients was collected between December 2019 and March 2020. A pretested self-designed tool was used for data collection. All sampled prescriptions were assessed for appropriateness using the STG of 2017 and WHO “AWaRe” classification. The criteria used in assessment included dose, frequency, duration of treatment and choice of antibiotic prescribed for disease condition. Descriptive statistics were used in data analysis.

**Results:**

a total of 184 patient prescriptions (286 antibiotics) were included in this study. Results showed that antibiotics were mostly prescribed for dental and dental-related conditions (20.7%) and obstetric post-delivery prophylaxis (18.1%). Appropriateness of indicators for antibiotics prescribed assessed ranged between 89.2% to 97.6%. The most frequently prescribed antibiotics were metronidazole (25.9%), amoxicillin with clavulanic acid (22.0%), amoxicillin (16.4%) and ciprofloxacin (10.1%). Based on WHO “AWaRe” classification, the “access” group of antibiotics (74%) was the most prescribed, followed by “watch” group (24%). There were no antibiotics prescribed from the “reserve” group of antibiotics and another 2% that was not part of AwaRe classification.

**Conclusion:**

study revealed that the level of appropriateness for prescribing indicators assessed was relatively high and majority of prescribed antibiotics were from the “access” and “watch” group. These observations suggest responsible prescribing of antibiotics at the Ghana Police Hospital and effective antibiotic stewardship should be sustained and improved.

## Introduction

Antibiotics have been used widely in the prevention and management of infections since the discovery of penicillin in the 1920´s. This discovery, among others, has changed the course of medicine and reduced infection-related mortality. In recent times, however, there have been concerns over the inappropriate use of antibiotics which has partly contributed to the development of resistance in disease-causing microorganisms [1].

Antimicrobial resistance (AMR) occurs when bacteria, viruses, fungi and parasites change over time and no longer respond to previously recommended antibiotics [2]. This makes it difficult to treat infections, thereby, increasing the risk of disease spread, severe illness and death. There are reports that suggest that *Acinetobacter species, Pseudomonas species, Escherichia coli, Klebsiella pneumoniae, Salmonella enterica, Staphylococcus aureus* and *Streptococcus pneumonia* have become resistant to commonly used antimicrobials [3,4]. Recent reports also suggest that some microorganisms have developed resistance to carbapenems, which are antibiotics often used as last resort for *K. pneumonia* infections [4]. It is estimated that approximately 700,000 deaths occur annually as a result of AMR and deaths from AMR could rise to 10 million by 2050 if no concerted action is taken [5]. The association between antimicrobial use and resistance has been well documented in a number of individual healthcare facilities, communities and countries. There is sufficient data to suggest association between AMR and the levels of antimicrobial use. Furthermore, recent studies have also revealed a general shift towards the use of broad-spectrum and last-resort antibiotics [6-8].

Globally, antimicrobial stewardship (AMS) programmes have evolved in response to AMR [9]. These AMS programmes are aimed at raising awareness of AMR, building capacity of health workers and promoting rational use of antibiotics, among others. The Ghana Police Hospital has benefitted from antimicrobial stewardship programmes via the Healthcare Improvement Scotland Partnership, part of the Commonwealth Pharmacists Antimicrobial Stewardship programme. Personnel within Ghana Police Hospital, primarily medical officers, pharmacists and nurses, have undergone AMS training. Campaigns to raise awareness on AMR have also featured prominently as part of the continuing education programmes for staff of the hospital. Ghana Police Hospital has also participated in the Global Point Prevalence Survey (PPS) programme for in-patient antimicrobial prescribing surveillance [9]. This research was a follow-up (pilot) on PPS with the aim of describing the antibiotic prescribing pattern for both in-patients and out-patients and also assessing the appropriateness of antibiotic prescribing within the hospital.

## Methods

**Study site:** the study was conducted at the Ghana Police Hospital, Accra, Ghana. The hospital is a 100-bed facility providing health services to police personnel, their dependents and general public. Ghana Police Hospital offers out-patient services and some specialty services which include medical, orthopaedics, obstetrics and gynaecology, ophthalmology, surgical, paediatric and public health. It also serves as a referral health facility during national crisis and disasters. The hospital provides out-patient services to over 100,000 patients annually. The hospital also has two pharmacies that ensure reliable supply of medicines throughout the year to patients. National Health Insurance Scheme (NHIS) subscribers receive medicines that are indicated as part of the scheme for free while non-subscribers have to pay for drugs.

**Study design:** a cross sectional descriptive study was employed. Quantitative data was collected and analyzed. Data collection for this study was carried out between December 2019 to March 2020.

**Focus of study:** this research focused mainly on the prescription pattern of antibiotics at the Ghana Police Hospital within the study period.

**Data source:** data was collected from prescriptions of out-patients and in-patients folders within clinical areas and at the hospital pharmacies.

**Eligibility criteria and ethical considerations:** all prescriptions with antibiotics irrespective of dosage form or age of patient were eligible for inclusion. The study excluded prescription from patients on human immunodeficiency virus and tuberculosis treatments. Also, prescriptions received from facilities outside the Ghana Police Hospital were excluded. The study protocol received approval from the Ghana Police Hospital administration. Patients´ data were handled with utmost confidentiality, and all patient and doctor identifiers were removed.

**Sampling, data collection and analysis:** a simple random sampling method was used in selecting patient prescriptions. Data primarily collected from patient folders/prescriptions included demographic information (age, gender etc.) using a self-designed instrument. Other variables that were collected included name of antibiotic, dose and duration of treatment, and the diagnosis for which the antibiotic was prescribed. Prior to its use, the data collection instrument was piloted using 20 prescriptions collected from the hospital pharmacy. Data collected was organized into out-patient antibiotic prescriptions and in-patient antibiotic prescriptions. All the prescriptions were assessed using the Ghana Standard Treatment Guidelines (2017) to determine appropriateness of antibiotics prescribed. The appropriateness of antibiotics prescribed, was determined using the following criteria; dose, frequency, duration of treatment and the choice (selection) of antibiotic prescribed for the disease condition diagnosed. Furthermore, the prescribed antibiotics were classified according to the World Health Organization (WHO) Access, Watch, Reserve (“AWaRe”) groupings to determine antimicrobial stewardship [10]. Data was analyzed using univariate methods such as frequency tables.

## Results

**Patients´ demographics:** a total of 184 patient prescriptions were included in this study. Majority of the prescriptions 60.9% (n=112) were for female patients whilst 39.1% (n=72) were for male patients. Prescriptions with antibiotics for out-patients constituted 72.8% (n=134), whilst that for in-patients was 27.2% (n=54).

Furthermore, 24.5% (n=45) of patients prescribed antibiotics were police officers, whilst 75.5% (n=139) were civilians. Patients aged 15 to 44 years received the highest number of prescriptions for antibiotics (57.6%). Further analysis of the age distribution for patients prescribed antibiotics revealed; children under one-year-old (1.1%), children between 1 to 14 years (16.3%), ages 45-64 years (15.8%), and patients 65 years old (9.2%).

**Indications of prescribed antibiotics:** conditions that required frequent antibiotic therapy include dental and related infections (tooth extraction, fractures, periodontitis, mandibular, and pulpitis). Obstetric post-delivery prophylaxis, respiratory tract infections (RTIs) and gastrointestinal tract infections were next most common indications for antibiotic treatment (Table 1). There were instances of comorbid infections for which antibiotics were prescribed in the other conditions (n<6).

**Table 1:** top ten conditions for which antibiotics were prescribed at the Ghana Police Hospital

Indications	Frequency (N)	Proportion (%N)
Dental and related infections	39	20.7
Post-delivery prophylaxis	34	18.1
Respiratory tract infections	26	13.8
Gastrointestinal tract infections	23	12.3
Urinary tract infections	17	9.0
Skin and soft tissue infections	12	6.4
Sexually transmitted infections	9	4.8
Caesarean section	6	3.2
Enteric fever	6	3.2
Others	16	8.5
Total	Σ *N*= 188	Σ%*N*= 100

**Commonly prescribed antibiotics:** metronidazole (25.9%) was the most frequently prescribed antibiotic. Other commonly prescribed antibiotics included co-amoxiclav (amoxicillin + clavulanic acid), amoxicillin, ciprofloxacin, and cefuroxime (Table 2). Some patients were prescribed more than one antibiotic. Least prescribed antibiotics during the study period included: secnidazole, clindamycin, azithromycin, mupirocin, fluconazole, cefotaxime, levofloxacin, ceftriaxone-sulbactam and gentamicin.

**Table 2:** commonly prescribed antibiotics at the Ghana Police Hospital

Indications	Frequency (N)	Proportion (%N)
Metronidazole	74	25.9
Amoxicillin + clavulanic acid	63	22.0
Amoxicillin	47	16.4
Ciprofloxacin	29	10.1
Cefuroxime	23	8.0
Flucloxacillin	10	3.5
Clarithromycin	8	2.8
Doxycycline	7	2.4
Ceftriaxone	5	1.7
Others	20	6.9
Total	Σ *N*= 286	Σ%*N*= 100

**Figure 1:**
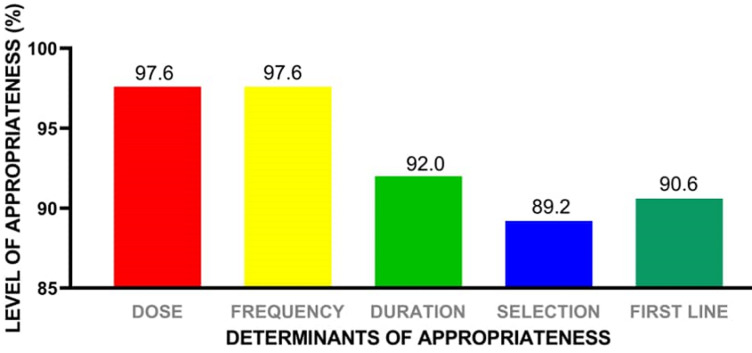
appropriateness of antibiotic prescribing indicators at the Ghana Police Hospital

**Prescribing indicators:** results obtained for the following indicators: dose, frequency of dosing (dosing interval), duration of therapy, among others are shown in Figure 1. The data showed that appropriateness for all indicators ranged from 89.2% to 97.6%.

**Antibiotic prescribing pattern based on WHO “AWaRe” classification:** of the 286 antibiotics reviewed, the “Access” group of antibiotics were the most prescribed, having a proportion of 74%, followed by the “Watch” group (24%). There were no antibiotics from the “Reserve” group of antibiotics. However, 2% of antibiotics prescribed such as; levofloxacin, mupirocin, and ceftriaxone-sulbactam combinations did not belong to any of the AWaRE classifications.

## Discussion

Antimicrobial resistance (AMR) remains a global public health threat. Regular monitoring and studies on antimicrobial prescribing (and use) in relevant sectors are critical to generate data and lead to information-based stewardship interventions. The current study revealed that more females (60.9%) received prescriptions with antibiotics than males (39.1%). Other studies corroborate this finding which suggests that female patients may be receiving more antibiotics than male patients [11-13].

Indications for which antibiotics were frequently prescribed in this study were similar to those observed in recent point prevalence studies in two Ghanaian Hospitals: Keta Municipal Hospital and Ghana Police Hospital [14]. Common indications for which antibiotics were prescribed in the aforementioned study included; obstetric/gynecological infections, respiratory tract infections; skin and soft tissue infections; ear, nose and throat infections; and bronchitis. However, the current study revealed that, patients who accessed dental care and related conditions were most frequently prescribed antibiotics. This observation could be due to the fact that this study captured both in-patients and out-patient cases whereas the point prevalence study [14] focused on only in-patients. Obstetric post-delivery antibacterial prophylaxis was the second most frequent condition for which antibiotics were prescribed in the current study. Routine antibiotic prophylaxis after caesarean section or operative vaginal birth has been shown to be effective, reducing the risk of confirmed systemic infections by 56% compared with placebo [15].

The WHO highly recommends antibiotic prophylaxis because of strong evidence suggesting that an estimated 16% of women have infection following operative vaginal birth [16]. Also, evidence from a systematic review indicates that maternal infections are reduced by 60-70% following antibiotic prophylaxis [17]. As a standard for prevention of infections in obstetric care, a combination of amoxicillin and metronidazole is recommended by the STG (2017) [18]. The antibiotics frequently prescribed for post-delivery antibacterial prophylaxis in this study were a combination of metronidazole and amoxicillin for a period of 5 to 7 days which was consistent with the hospital developed guidelines, and also recommendations by the WHO [19]. The ‘after operative vaginal delivery´ (ANODE) trial, however, recommends a single dose of prophylactic antibiotics, such as intravenous co-amoxiclav, after operative vaginal delivery or birth [15].

Commonly prescribed antibiotics in this study were similar to those observed in point prevalence studies in Ghana [11,14]. Among the prescribed antibiotics in this study were; penicillins and other β-lactam antibiotics, combinations of penicillins with a β-lactamase inhibitor (co-amoxiclav), second- and third-generation cephalosporins, quinolones, macrolides, aminoglycosides, among others. Metronidazole was found to be the most frequently (25.9%) prescribed antibiotic at the Ghana Police Hospital, and tends to conform with the observation that it is the most used antibiotic in Ghana, and marketed under several brand names [20].

Metronidazole was prescribed mostly in combination with amoxicillin in obstetrics and gynaecological care (mainly in post-delivery prophylaxis), prophylaxis in dental related conditions, *H. pylori* eradication, and occasionally in caesarean sections. Antibiotics used in dental related cases such as tooth extraction, irreversible pulpitis, and periodontitis in this study were mainly metronidazole, amoxicillin and co-amoxiclav. Sturrock *et al*. in an audit review of antibiotic prescriptions by dental practitioners, found that amoxicillin (61.2%) was the most frequently prescribed antibiotic followed by metronidazole (29.9%) [21]. Similarly, Ford *et al*. also found that in Australia, amoxicillin was the most prescribed antibiotic by dental practitioners, although there was inappropriate prescribing of amoxicillin sometimes [22]. The second most prescribed antibiotic was co-amoxiclav, effective against infections (caused by beta-lactamase-producing bacteria strains) like RTIs, bone and joint infections, among others [23]. Penicillins are the recommended drug for treating acute RTIs in Ghana [18], as against the use of third generation cephalosporins in Japan [24].

In this study, antibiotics were used frequently for RTIs such as tonsillitis, pharyngitis, pneumonia, among others. Cefuroxime was prescribed for RTIs such as pneumonia, particularly in children between 1-14 years, and in some mild cases of urinary tract infections (UTIs). These prescriptions were found to be appropriate for disease conditions [18,23]. Other antibiotics used for RTI in this study were azithromycin and amoxicillin. Ciprofloxacin was prescribed often for management of enteric fever, some UTIs such as cystitis and the management of sexually transmitted infections such as gonorrhoea. Oral flucloxacillin (a penicillinase-resistant penicillin) which has activity against penicillin-resistant staphylococci [23], was often prescribed for treatment of skin and soft tissue infections; which included impetigo and boils in children. Flucloxacillin was also used in combination with ceftriaxone in the management of cellulitis.

Sub-therapeutic doses of antimicrobials have been found to be important in development of antimicrobial resistance in disease-causing microorganisms [25]. In this study, relatively high level of appropriateness of antibiotics prescribed per the dose, frequency, duration of treatment, and selection of antibiotic for disease condition were observed as values obtained ranged between 89.2% to 97.6%. Treatment guidelines for majority of the common infections among the outpatients available in the STGs, recent AMS interventions and regular staff education on AMR in the hospital could be contributing factors to this observation. Antimicrobial stewardship (AMS) interventions such as training and monitoring of prescribed antibiotics should be sustained to optimize antimicrobial use in hospitals. The STG (2017), makes recommendations for selection of drugs as either first line or second line for the management of various infections and diseases [18].

This study found that, 90.6% of antibiotics prescribed were consistent with recommended first line treatment options. For example, for oral and dental conditions such as periodontitis, clindamycin oral or amoxicillin/clavulanic acid is the recommended first line option for treatment, and findings from this study showed appropriateness in the prescribing patterns as recommended by the STG [18]. Related studies in South Africa and in United States of America have showed that some antibiotics were prescribed inappropriately by dental practitioners and did not follow any standard guidelines [26,27]. In the current study, ciprofloxacin was mostly prescribed as first line option for treatment of enteric fever as recommended by the STG [18].

The WHO “AWaRe” group classification of antimicrobials is to ensure more responsible prescribing with increased use of recommended antibiotics on the Access list, and reducing the use of antibiotics on the Watch and Reserve lists [25]. The WHO estimates that prescribing more than 60% of all antibiotics from the Access group would ensure easy access to basic antibiotics, reduce risk of antibiotic resistance and promote responsible use of antibiotics [10]. This level of Access antibiotics (74%) prescribed was very significant because it was higher than the WHO target of 60% by 2023. In this study, some of the antibiotics prescribed in “Access” class were metronidazole, amoxicillin, co-amoxiclav, flucloxacillin and clindamycin, among others. Antibiotics prescribed from the “Watch” group in this study was approximately 24%, which was much lower than the 47% obtained in a related study from three hospitals in Ghana [11].

This could be attributed to majority of the patients in this study being out-patients compared to the latter which had only in-patients. In-patients are likely to have moderate to severe infections compared to out-patients. Some of the “Watch” antibiotics prescribed at the Ghana Police Hospital included azithromycin, ciprofloxacin, ceftriaxone and cefuroxime. The study also revealed that, there were no antibiotics prescribed from the list of “Reserve” group similar to related studies in Ghana [11,28]. This suggests responsible prescribing of antibiotics at the Ghana Police Hospital, as earlier studies also indicate [28,29]. Although there was high level of appropriateness in antibiotic prescribing in this study with reference to guidelines, the study did not determine clinical appropriateness of antibiotics at individual patient level.

## Conclusion

Antibiotics were mostly prescribed for dental and dental-related infections, and for prophylaxis against infections in obstetrics and gynaecological care. The relatively high level of compliance with prescribing indicators for antibiotics was encouraging. Regarding the WHO AWaRe classification, majority of prescribed antibiotics were in the “Access” group, with only about a quarter in the “Watch” group, with no prescribed antibiotics in the “Reserve” group. It is recommended that regular antimicrobial stewardship interventions be conducted in hospitals or healthcare facilities to help minimize level of inappropriate antibiotic prescribing and consolidate antimicrobial stewardship interventions.

### What is known about this topic

- Inappropriate prescribing and use of antibiotics often leads to antimicrobial resistance;
- Adherence to national treatment guidelines is a key antimicrobial stewardship intervention to achieve desired therapeutic outcomes and minimize antimicrobial resistance.

### What this study adds

- Health personnel can attain the WHO target for prescribing antibiotics with well laid out antimicrobial stewardship programmes;
- High level of compliance with prescribing indicators for antibiotics is achievable.
